# Optimizing patient-reported sociodemographic measures in health care – a national example of the development and evaluation of a practical standardized instrument for collecting patient data in studies

**DOI:** 10.1186/s12874-026-02977-z

**Published:** 2026-08-20

**Authors:** Sigrid Boczor, Heike Hansen, Thomas Kloppe, Claudia Mews, Cathleen Muche-Borowski, Anja Rakebrandt, Ingmar Schäfer, Nadine Janis Pohontsch, Vivien Böttcher, Thorben W. Fründt, Anna Sophie Hoffmann, Jan Dietrich Philipp Köster, Martin Scherer

**Affiliations:** 1 Department of General Practice and Primary Care, University Medical Center Hamburg-Eppendorf, Martinistraße 52, Hamburg, 20246 Germany; 2 Department of Medicine, University Medical Center Hamburg-Eppendorf, Martinistraße 52, Hamburg, 20246 Germany; 3 Department of Otorhinolaryngology, University Medical Center Hamburg-Eppendorf, Martinistraße 52, Hamburg, 20246 Germany

**Keywords:** Sociodemographic data, Patient study data, Patient instrument, Survey, Operationalization, Data standardization, Evaluation, Evidence-based medicine

## Abstract

**Background:**

The development of patient-oriented health care is continuous subject to old and new challenges. To better identify patient needs, studies are necessary and standardized data collection is essential to describe, analyze and compare data. Associated demographic variables remain a topic of discussion in health services research. Common approaches either differ in relevant sociodemographic aspects, or their standardization is too detailed to be feasibly used in studies with a clinical or health services research focus. Our aim was to develop and evaluate a comprehensive and practical instrument for collecting sociodemographic patient data in studies.

**Methods:**

A variant (interdisciplinary expert panel) of the nominal group technique was used as a consensus method, in addition, a focus group was held and a 2-phase pretest according to Prüfer and Rexroth (method: “Think Aloud”) was used for evaluation. In the first phase we conducted 6 interviews with potential patients. The main phase was planned to enroll at least 200 patients to test the instrument with a quantitative sample as recommended for testing patient-reported outcome measures. It took place in eight departments of the University Medical Center Hamburg-Eppendorf. Exploratory analyses were conducted using SPSS. The interdisciplinary expert panel revised the instrument.

**Results:**

In the main phase, 290 (51%) of 574 approached patients participated [median (minimum; maximum) age: 40 (19; 86) years; 55% female]. Of these respondents 66%/ 31%/ 3% considered the length of the questionnaire to be appropriate/ too long/ too short; 96% found it understandable. Seven questions on living and income situation (commented at least three times) showed a need for clarification and modification. Non-responders optionally provided reasons for decline of which 7 clusters could be formed (predominantly: time). Final standardized variables: biological sex; year/country of birth; marital status/biological children; living/household type/persons/age; highest school/ qualification/current profession; household income/specialties/persons; country of birth mother/father; nationalities/residence status/native languages/German skills.

**Conclusions:**

Patient involvement is helpful in the development of patient survey instruments. Our instrument is based on the patients’ perspective and understanding of these measurements. The aim is to further strengthen patient-centered health care. The revised instrument will be used in studies. The expert group will continue to identify and implement socio-demographic developments.

**Supplementary Information:**

The online version contains supplementary material available at 10.1186/s12874-026-02977-z.

## Disclaimer

Initial results of this study were presented at the 25th Annual Conference of the Evidence-based Medicine Network (EbM Congress 2024) under the motto: Evidence-based policy and health care - an achievable goal or an illusion? 

## Introduction

The development of patient-oriented health care is continuous subject to old and new challenges like addressing health disparities, the recognition of underrepresented population groups, and demographic change [1–5]. In order to better identify patient needs, studies are necessary and a standardized data collection is essential to describe, analyze and compare data to facilitate statistical analysis of the association between sociodemographic data and health (problems) [6–8]. As a prerequisite for applicability, evidence-based medicine should routinely be guided by sociodemographic comparable samples and correspondingly operationalized moderator variables to adjust analyses [9, 10]. However, demographic variables in health care research remain a topic of actual debate [11–13]. Current commonly used approaches either differ in relevant sociodemographic aspects or their standardization is too detailed to be feasibly used in studies with a clinical or health care research focus [14, 15]. For instance, comparability at European level between the so-called microcensus surveys of the population is possible due to an EU-wide harmonized questionnaire [16]. However, the core program within the “Income and Living Conditions” section of the 2023 Microcensus comprises 252 questions regarding corresponding sociodemographic characteristics for persons up to 15 years of age, and as many as 333 questions from this age onwards [16]. In research, for example, Lotfaliany et al. 2024 presented their Australian questionnaire on sociodemographic and clinical questions on mental health [17]. On the one hand, by relying on a literature review including several randomized controlled trials in their field of research, the authors refer to a variety of clinical trials [17]. On the other hand they do not include observational studies in the wide field of health services research [17]. These examples show the ongoing need for clarification and harmonization of sociodemographic data assessment in studies in different fields of research. A consequence of using different approaches (with different definitions of possible moderator variables) concerns the quality of recommendations [18]. The latter require more efforts to search for possible significant differences in the conduct of the studies and to clarify any observed discrepancies in results before recommendations for everyday practice can be made [18]. This challenge cannot be solved by meta-analyses, unless they result in a loss of quality [18]. In contrast to existing approaches, our study uses an evidence-based mixed methods approach to resolve these obstacles. We have set up an interdisciplinary expert panel at the Department of General Practice and Primary Care at the University Medical Center Hamburg-Eppendorf to develop and evaluate a comprehensive yet practical instrument for collecting sociodemographic patient data in studies.

## Methods

### Methodological design

**Fig. 1:**
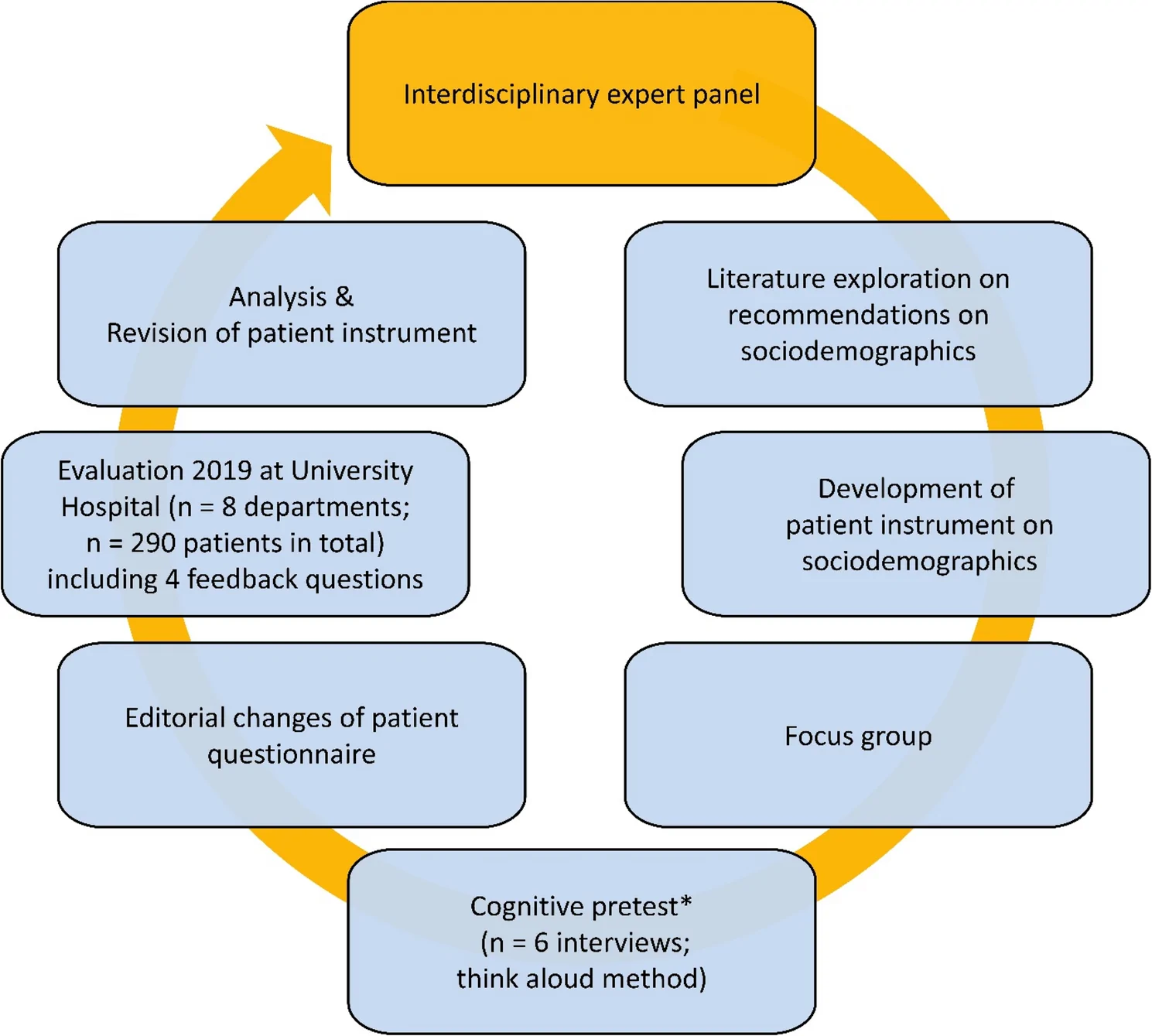
The individual steps of the presented development and evaluation of scientific recommendations for the standardized assessment of sociodemographic data. *A 2-phase-pre-testing according to Prüfer & Rexroth [20] was used

Following suggestions described in the framework for developing and evaluating complex interventions of the medical research council [19], the development and evaluation of the instrument was performed as multi-stage process. Accordingly, we conducted a mixed method study including a focus group, qualitative interviews and a quantitative survey. The individual steps of the development and evaluation are visualized in Fig. 1.

The development of the presented standardized questionnaire began with the formation of an interdisciplinary expert panel at the Department of General Practice and Primary Care at the University Medical Center Hamburg-Eppendorf in Germany. As a first step, the expert panel explored the literature regarding existing recommendations for the collection of sociodemographic data. In addition, surveys from large, published studies were examined. Based on the sources explored, sociodemographic variables as well as various methods for their assessment were discussed. A pre-final patient instrument on sociodemographics was developed. The pre-final questionnaire was discussed in a specially convened group of other experienced scientists at the institute. Subsequently, cognitive pre-tests were performed. This led to editorial changes of the pre-final instrument. Then the resulting patient instrument was evaluated in eight departments of the university hospital, including four feedback questions. The evaluation involved 290 participating patients, whose responses to the sociodemographic questions were analyzed. A revision of the instrument followed. In the following, the development and evaluation are described in greater detail, and the results are presented and discussed.

### Sociodemographic components

Referring to Grant and Booth 2009 [21], the primary compilation, in order to prioritize the discussion, was focused on a thematic literature overview of the sociodemographic bases. These included the common German joint recommendations from the Federal Statistical Office of Germany (DESTATIS) [21–23]. Further, surveys of a variety of established German socio-economic and social science research institutes [20, 24–26] and large German population surveys. These included, in particular, the MONItoring trends and determinants in CArdiovascular diseases (MONICA)/ Cooperative Health Research in the Region of Augsburg (KORA) cohort study which was part of the multinational World Health Organization’s (WHO) MONICA project [27, 28]. Furthermore, the Socio-Economic Panel (SOEP) [29–32], the periodically repeated German Health Interview and Examination Survey for Adults (DEGS) [33, 34], and the German National Cohort (GNC; German: NAKO) [35].

As consensus method a variation of the nominal group technique also referred to as expert panel [22–24] was used. Components included in the questionnaire were chosen by majority vote in the interdisciplinary expert panel, based on the long study experience of the panel members. This development included: (1) Generating ideas and suggestions for items to be included in the instrument. Subgroups by specializations within the interdisciplinary expert panel were formed for the collected items to discuss and develop suggestions for the assessment of items in detail. (2) Clarification and ranking of items including individual variable definitions and respond categories were discussed and tentatively determined in recurring subgroup meetings up to one to two hours. (3) The subgroups presented their findings to the entire interdisciplinary expert panel in meetings lasting between one and two hours. During these meetings, the findings were re-evaluated by the entire panel following in-depth discussion. Any rewording or regrouping of items required previous considerations to be taken into account once again. In cases involving more complex discussions, the task of revising the findings was returned to the respective subgroup. (4) This process continued until consensus was agreed upon and, if no unanimous vote could be reached after repeated discussions, determined by majority vote at meetings of the entire interdisciplinary expert panel. The developed and evaluated instrument comprised of 21 questions on the sociodemographic aspects of sex, age, type of residence, marital or partnership status, household size and structure, education, vocational training, income and migration-related determinants; four additional feedback questions were asked in both pretest phases about how the instrument was accepted and understood [see Additional file 1].

### Interdisciplinary expert panel and focus group members

The interdisciplinary expert panel was initiated with seven members, the majority of whom are research associates, with long-term experience in the design and conduct of quantitative clinical and observational or interview studies. One of whom is a medical doctor, the other members are specialists in medical guideline development, medical documentation, data management and/or statistical analyses including two researchers with focus on health economics and social work (quantitative and interview analyses).

With respect to the standardization of the subsequent conduct of interviews for the evaluation, the interdisciplinary expert panel later invited an expert for psychology and qualitative study methodology. It was decided on a 2-phase pretest according to Prüfer and Rexroth [20]. Before starting the 2-phase pretest, it was further decided to discuss the preliminary version of the questionnaire in an extra one-hour long focus group. The focus group was held with four additional experienced research associates of the Department of General Practice and Primary Care (three psychologists and one medical doctor with longstanding research experience in health care research) who were not members of the interdisciplinary expert panel to gain initial feedback. The questionnaire was presented to the focus group participants in its pre-final form. They were asked whether the questionnaire could be used as it stood and what barriers they expected study participants or patients would encounter when completing it. Important points of discussion were recorded in written minutes.

### The cognitive interviews

The first pretest phase included six cognitive interviews, conducted in line with recommendations from Frost et al. 2007 [25]. Cognitive interviews are used in questionnaire development to assess and optimize the comprehensibility, interpretation preferences and thought patterns of respondents regarding a questionnaire. They serve to identify potential misunderstandings, interpretation problems and barriers to completion and to increase the validity of the instrument [26]. The “Think Aloud” method was used [27], which means that participants were observed while filling out the questionnaire independently and motivated to think aloud about answering the questions. The interviewers asked specific follow-up questions (a cognitive interview technique known as verbal probing [26]) if, for example, they noticed hesitation in answering questions or if the answer to a question was changed. Interviews were conducted in personal environment (family members, acquaintances, or neighbors) by some of the interdisciplinary expert panel members experienced in qualitative research. Interviews included written informed consent and were selected to cover the following factors: wide age range (20–70 years of age), sex (female/male), educational level (high school diploma yes/no), migration-related (yes/no).

To minimize the obstacles to the inclusion of interview participants and the implementation of the interviews, and to ensure the anonymity of the respondents, it was decided not to make audio recordings. The interviews were documented by the interviewer using a standardized documentation form and the results were discussed in the interdisciplinary expert panel.

### The evaluation study

The second pretest phase, i.e. the main evaluation study took place in November and December 2019 in the waiting areas of the eight participating departments of the University Medical Center Hamburg-Eppendorf (Table 1); Recruitment times (day of the week and time) were generally coordinated with but not prespecified by the departments and depended on the medical consultation hours offered and availability of study assistance.

**Table 1:** The participating departments with their outpatient waiting areas (in alphabetical order) that took part in the evaluation study. All departments are located at the University Medical Center Hamburg-Eppendorf, Hamburg, Germany

Specialty department
I. Department of Medicine (Gastroenterology with the Sections Infectiology and Tropical Medicine): Initial presentation for liver disease
III. Department of Medicine (Nephrology/Rheumatology with Section Endocrinology)
Department of Cardiology
Department of Diagnostic and Interventional Radiology and Nuclear Medicine
Department of Otorhinolaryngology: Tumor follow-up consultations statutory
Department of Periodontics, Preventive and Restorative Dentistry
Department of Trauma and Orthopedic Surgery
Outpatient Center UKE-Medizinisches Versorgungszentrum (MVZ)/ General Medicine

This study was not a randomized controlled trial. A cluster randomization by department was not performed. Nevertheless, the objective was to achieve the most balanced distribution possible across the various departments. This aim served to avoid potential biases stemming from specialty-specific or external differences in characteristics — biases that could be reflected both in patients’ responses to the instrument and in their answers to the feedback questions [28].

Patients who gave written informed consent were motivated using the think-aloud method when completing the questionnaire in a voice appropriate to the situation in the waiting area, if possible; otherwise, appropriate written comments were requested. The remaining waiting time until the medical consultation could limit the completion of the questionnaire. In the case of non-responders, a reason for decline, optionally stated by the patient, could be noted in the list used to count the patients that were approached, participating and declining.

### Sample size

The goal for the quantitative evaluation study was to include at least 200 participating patients, as recommended for testing patient-reported outcome measures by Frost et al. 2007 [25]. Based on the eight participating departments, the target was to enroll 25 patients per department, and we set the recruitment period for 2 months. A student assistant kept a list of how many patients in each waiting area participated in or declined our evaluation study. Participants received no incentive for participation.

### Study population

**Fig. 2:**
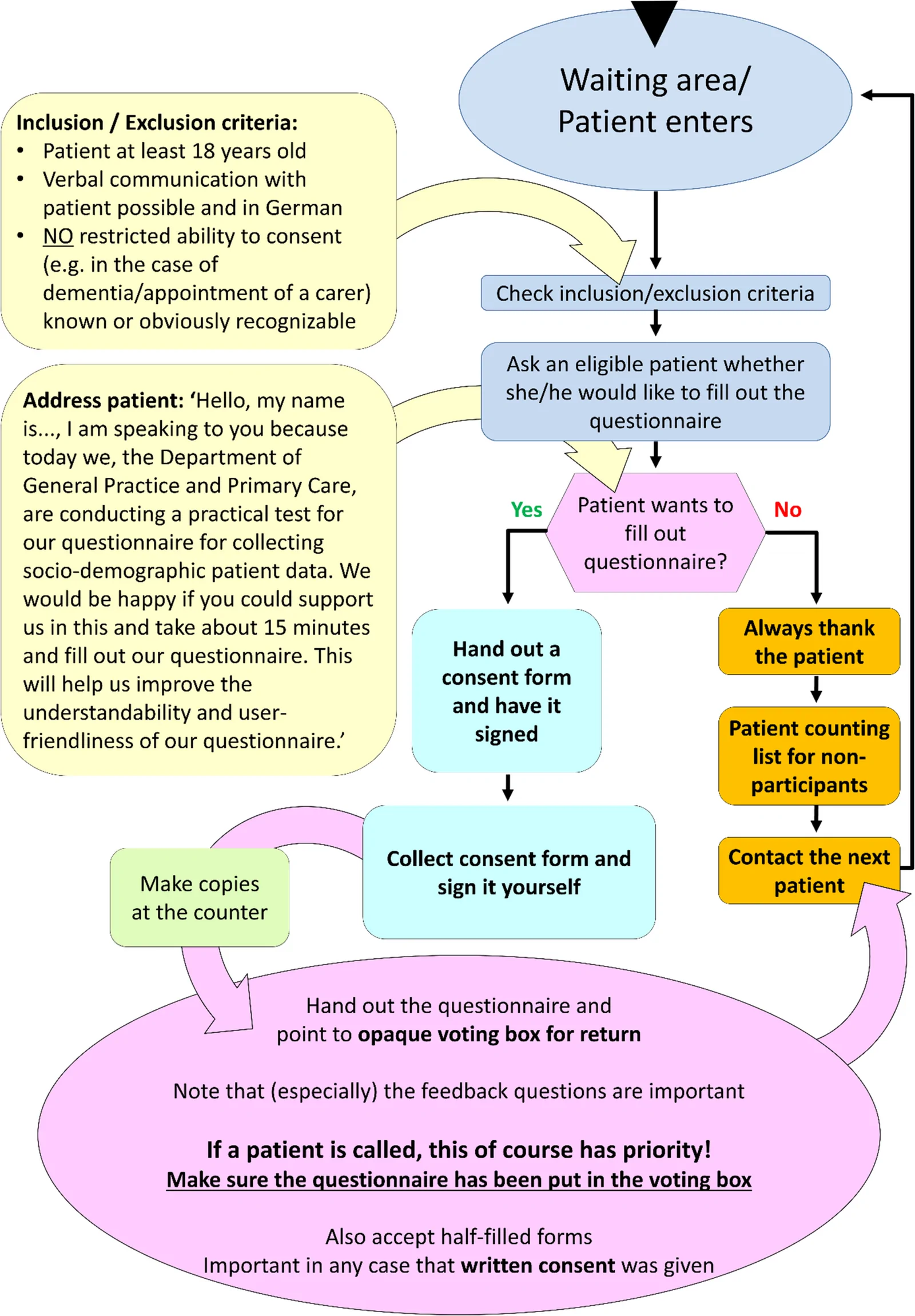
The standardized procedure for the evaluation study

In the evaluation study, patients in the waiting areas were consecutively approached by a student assistant following a standardized workflow (Fig. 2); Besides written informed consent, inclusion criterion was an age of at least 18 years; exclusion criterion, respectively, was insufficient language skills to understand and answer the questionnaire.

### Data management and analysis

Data from the patient instrument and answers given to the feedback-questions were manually entered using data masks created with EpiData 4.6.0.0 and exported. The exploratory data analyses were performed by IBM SPSS 29 and SPSS 25 for MacOS. The frequencies of decline compared to acceptance of participation in the evaluation study were compared by Pearson chi-squared test. Descriptive statistics of the participants of the evaluation study include absolute and relative frequencies for the categorical variables (marital status, having children, living and housing situation, highest school and education degree, current profession, migration-related determinants). For the continuous non-normally distributed variables the median with minimum and maximum is given, i.e. for year of birth, calculated age at year of the evaluation survey, number of children, and number of persons in household categorized following the guidelines at the time in < 15/15–18/>18 years. The monthly equalized disposable income of households in euros was calculated by dividing the average value of the respective category (< 700; 700 – <900; 900 – >1100; 1100 – <1300; 1300 – <1500; 1500 – <2000; 2000 – <2500; 1500 – <3000; 3000 – <3500; 3500 – <4000; 4000 – <5000; 5000 – <6000; 6000 – <7500; 7500 – <10000; 10000 – <18000; ≥ 18000) by the equivalent household size according to the so-called modified Organisation for Economic Co-operation and Development (OECD) equivalence scale ([29]; see also Table 3); this calculation takes into account both the number of persons and the age structure of a household through a needs weighting. All measures were calculated for total sample as well as splitted by sex. For data protection regulations, the results were not broken down by departments involved. For patients who declined to participate but optionally wanted to provide a reason for non-participation, this reason was noted and categorized and reported descriptively with absolute frequencies.

The pretest analyses and the subsequent second revision of the instrument took place in 2023. Basic methodology framework and initial results were presented at the 25th Annual Conference of the Evidence-based Medicine Network (EbM Congress 2024, Berlin, Germany) [30].

## Results

### The cognitive interviews

The 6 interviewees had a median (minimum; maximum) age of 41 (31; 68) years (50% female). The cognitive interviews resulted in the following editorial changes of the instrument: (1) The table of contents has been removed and the short subheadings for the sections have been inserted directly into the instrument; (2) The item headings “sex” and “year of birth” were replaced by fully worded questions addressing the participant directly (like with the other items); (3) The item “living alone” was split with a jump question so that the other answer options are only asked in the case of a negative answer. Additionally, one interviewee noted that although the length of the instrument was just right, it might be too long when combined with another study questionnaire.

### The evaluation study

#### Sociodemographic data of participants

**Fig. 3:**
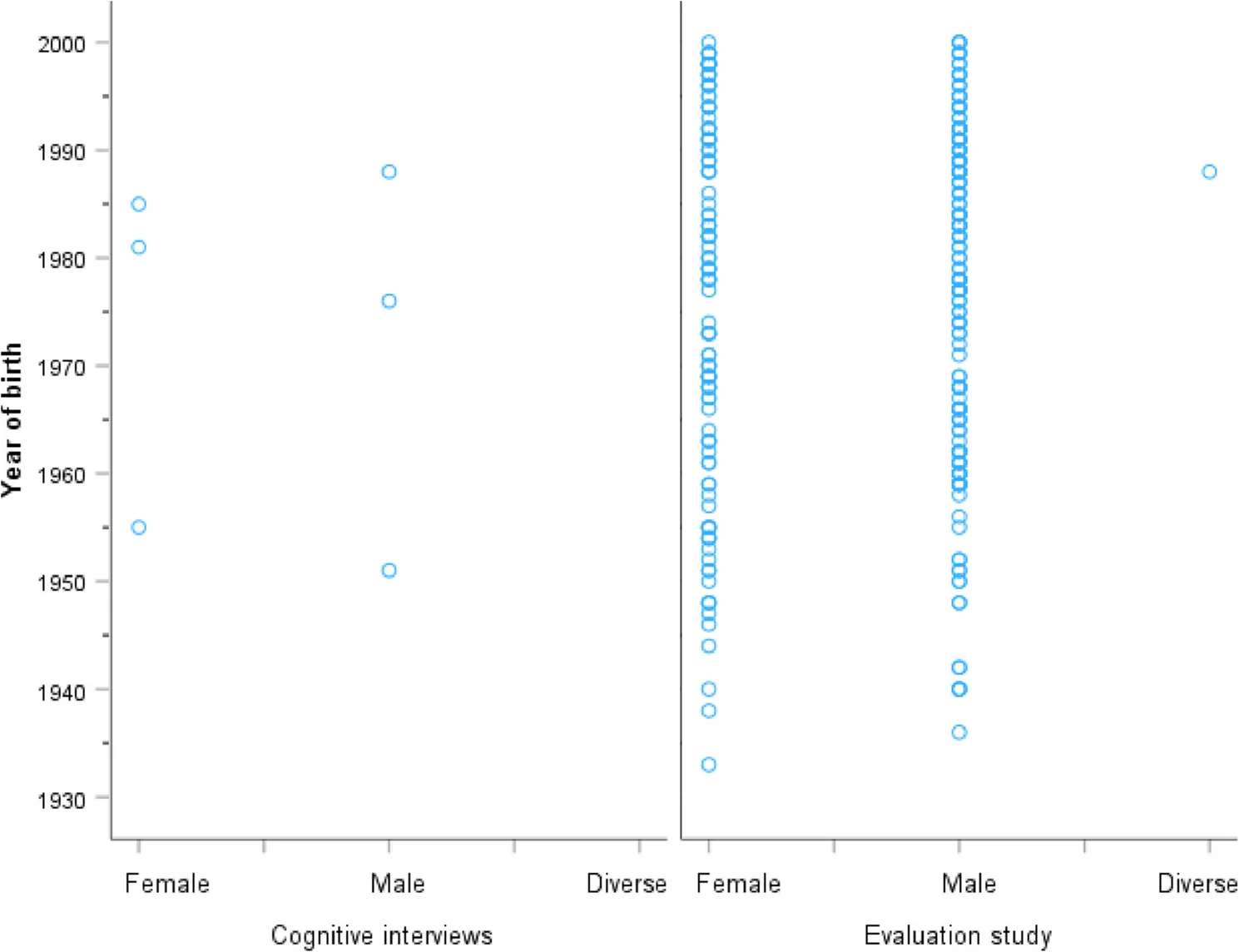
Age and sex of the participants of the evaluation study (*n* = 290) and cognitive pretests (*n* = 6)

Of 574 approached patients, 290 (51%) participated in the evaluation study with a median (minimum; maximum) age of 40 (19; 86) years, thereof 55% were female. Figure 3 illustrates the age and gender distribution of the participants in the cognitive interviews and the evaluation study.

The sociodemographic data of the evaluation studies participants are presented in detail in Tables 2 and 3.

**Table 2:** Descriptive statistics of the sociodemographic patient data provided in the evaluation study

Parameter	Statistical measures^1^ by patient sex and in total			
	Female *N* = 129 (44.5%)	Male *N* = 160 (55.2%)	Diverse *N* = 1 (0.3%)	Total *N* = 290
Year of birth, Median (minimum; maximum)	1933 (1978; 2000)	1936 (1978; 2000)	1988	1978 (1933; 2000)
Missing	0	0		0
Age, Median (min.; max.), ^2^	40 (19; 86)	40 (19; 83)		40 (19; 86)
Marital status				
Unmarried	61 (47.3%)	74 (46.3%)	1 (100%)	136 (46.9%)
Married	40 (31.0%)	47 (29.4%)		87 (30.0%)
Divorced	11 (8.5%)	28 (17.5%)		39 (13.4%)
Widowed	17 (13.2%)	11 (6.9%)		28 (9.7%)
Missing	0	0		0
Children^3^				
No	80 (62.0%)	97 (60.6%)	1 (100%)	178 (61.4%)
Yes	49 (38.0%)	63 (39.4%)		112 (38.4%)
Missing	0 (0%)	0 (0%)		0 (0%)
If yes, median number of children (min.; max.)	2 (1–5)	1 (1–4)		1 (1–5)
Missing	0	1 (0.6%)		1 (0.3%)
Living situation				
Living alone				
Yes	37 (28.7%)	63 (39.4%)	1 (100%)	101 (34.8%)
No	92 (71.3%)	96 (60.0%)		188 (64.8%)
Missing	0 (0%)	1 (0.6%)		1 (0.3%)
If no, ^4^				
With Partner	44 (47.8%)	58 (60.4%)		102 (54.3%)
With children	31 (33.7%)	28 (29.2%)		59 (31.4%)
With Parents/ In-laws	17 (18.5%)	19 (19.8%)		36 (19.1%)
With other family member/s	16 (17.4%)	13 (13.5%)		29 (15.4%)
With other person/s	25 (27.2%)	17 (17.7%)		42 (22.3%)
Missing	1 (0.8%)	1 (0.6%)		2 (0.7%)
Housing situation				
Flat, rented	58 (45.0%)	75 (46.9%)		133 (45.9%)
House, rented	15 (11.6%)	15 (9,4%)		30 (10.3%)
In your own flat	22 (17.1%)	32 (20.0%)		54 (18.6%)
In your own house	20 (15.5%)	26 (16.3%)	1 (100%)	47 (16.2%)
Assisted living	8 (6.2%)	2 (1.3%)		10 (3.4%)
Old people’s/seniors’ home	6 (4.7%)	6 (3.8%)		12 (4.1%)
Nursing home “all-round care”	0	0		0
Other	0	2 (1.3%)		2 (0.7%)
Missing	0	2 (1.3%)		2 (0.7%)
Number persons in household in total^5^, Median (min.; max.)				
Incl. own				
person/children	2 (1–8)	2 (1–8)	1	2 (1–8)
Missing	1 (0.8%)	3 (1.9%)		4 (1.4%)
Thereof, number …, Median (min.; max.)				
Under 15 yrs.	0 (0–3)	0 (0–2)		0 (0–3)
15–18 yrs.	0 (0–2)	0 (0–2)		0 (0–2)
Above 18 yrs.	2 (1–7)	2(1–6)		2 (1–7)
Missing	1	1	0	2
Highest school degree				
No degree	10 (7.8%)	4 (2.5%)		14 (4.8%)
Secondary school/VHS^6^/ 8-year primary school	5 (3.9%)	7 (4.4%)		12 (4.1%)
Middle school leaving certificate/ secondary school/POS^7^	22 (17.1%)	35 (21.9%)		57 (19.7%)
University entrance qualification	10 (7.8%)	17 (10.6%)	1 (100%)	27 (9.3%)
A-levels	81 (62.8%)	95 (59.4%)		177 (70.0%)
Missing	1 (0.8%)	2 (1.3%)		3 (1.0%)
Highest education level				
No qualification	41 (31.8%)	26 (16.3%)		67 (23.1%)
Vocational-industrial training	20 (15.5%)	21 (13.1%)		41 (14.1%)
Vocational and school training	11 (8.5%)	17 (10.6%)		28 (9.7%)
Technical school/Master school/etc.	10 (7.8%)	25 (15.6%)		35 (12.1%)
University of Applied Sciences	4 (3.1%)	8 (5.0%)		12 (4.1%)
University	42 (32.6%)	58 (36.3%)	1 (100%)	101 (34.8%)
Missing	1 (0.8%)	5 (3.1%)		6 (2.1%)
Current profession, ^4^				
Pupil/ student/ trainee/ retraining	36 (21.6%)	31 (16.9%)		67 (21.2%)
Full time	26 (35.6%)	75 (40.8%)		101 (32.0%)
Part time	32 (19.2%)	25 (13.6%)		57 (18.0%)
Hours/week	MW: 22.0; SD 4.9	MW 21.6; SD 6.5		MW: 21.8; SD: 5.6
Self employed	12 (7.2%)	23 (12.5%)	1 (100%)	36 (11.4%)
Housewife/husband	28 (16.8%)	3 (1.6%)		31 (9.8%)
Unemployed	7 (4.2%)	9 (4.9%)		16 (5.1%)
Senior citizen	22 (13.2%)	14 (7.6%)		36 (12.7%)
other	4 (2.4%)	4(2.2%)		8 (2.5%)
Missing	1 (0.8%)	6 (3.8%)		7 (2.4%)
Monthly equalized disposable income ^8^, Median (min.; max.) in Euro	2166,67€ (350;18000)	2500,00€(1000;14000)	5500€	2250,00€(350;18000)
Missing	56 (56.6%)	54 (33.8%)		110 (37.9%)
Equalized household size ^8^, Median (min.; max.)	1.5 (1.0; 4.5)	1.5 (1.0; 4.3)	1.0	1.5 (1.0; 4.5)
Missing	2 (1.6%)	4 (2.5%)		6 (2.1%)
Migration-related determinants				
Country of birth	115 (89.1%)	133 (83.1%)		249 (85.9%)
Germany	10 (7.8%)	18 (11.3%)	1 (100%)	28 (9.7%)
Other	4 (3.1%)	9 (5.6%)		13 (4.5%)
Missing				
Living in Germany Since birth	114 (88.4%)	130 (81.3%)	1 (100%)	245 (84.5%)
Since year (min; max)	8 (6.2%; 1974; 2013)	18 (11.3; 1954; 2017)		26 (9.0%; 1954; 2017)
Missing	7 (5.4%)	12 (7.5%)		19 (6.6%)
Mother’s country of birth				
Germany	94 (72.9%)	115 (71.9%)	1 (100%)	210 (72.4%)
Other	29 (22.5%)	33 (20.6%)		62 (21.4%)
Missing	6 (4.7%)	12 (7.5%)		18 (6.2%)
Father’s country of birth				
Germany	81 (62.8%)	99 (61.9%)	1 (100%)	181 (62.4%)
Other	41 (31.8%)	49 (30.6%)		90 (31.0%)
Missing	7 (5.4%)	12 (7.5%)		19 (6.6%)
Native language is German, ^4^				
Yes	111 (86.0%)	130 (81.3%)	1 (100%)	242 (83.4%)
Other native lang.	18 (14.0%)	27 (16.9%)	0	45 (15.5%)
Missing	7 (5.4%)	13 (21.7%)		20 (6.9%)
*If Yes*, German lang. skills				
Very good	15 (11.6%)	14 (8.8%)		29 (10.0%)
Good	1 (0.8%)	2 (1.3%)		3 (1.0%)
Bad	0	1 (0.6%)		1 (0.3%)
Missing	113 (87.6%)	143 (89.4%)		257 (88.6%)
German citizenship				
Yes	121 (93.8%)	142 (88.8%)	1 (100%)	264 (91.0%)
No	2 (1.6%)	4 (2.5%)		6 (2.1%)
Missing	6 (4.7%)	14 (8.8%)		20 (6.9%)

*Yrs.* Years of age, *MW* mean, *SD* Standard deviation

^1^Absolute and relative frequencies if not stated otherwise

^2^Years calculated by given year of birth and year of evaluation (2019)

^3^Biological children or children for whom you take responsibility

^4^Multiple answers were allowed

^5^Constantly, including children and yourself

^6^Volkshochschule (Community College)

^7^Polytechnische Oberschule (ten-year general polytechnic secondary school in the former German Democratic Republic/GDR)

^8^Calculated using the modified OECD (Organisation for Economic Co-operation and Development)-scale weighting at state-of-the-art during time of evaluation study: 1.0 [own person] + (total number of persons in the household - number of persons under 15 years of age in the household - 1) * 0.5 + number under 15 years of age * 0.3; The standard age threshold for weighting for children had later been set at 14 years and the instrument was adapted accordingly (Statistical office of the Europenean Union. Eurostat Statistics Explained. Glossary: Equivalised disposable income. https://ec.europa.eu/eurostat/statistics-explained/index.php?title=Glossary:Equivalised_disposable_income. Accessed 15 Jul 2024.)

**Table 3:** List of other countries of birth and native languages that were specified in the evaluation study of patients with migration-related determinants

Parameter	Absolute frequencies^1^ by patient sex^2^ and in total		
	Female *N* = 10	Male *N* = 18	Total *N* = 28
Other country of birth,own person	Türkiye 3	Egypt 2	Türkiye 5
	Russia 2	Türkiye 2	Russia 3
	Bulgaria 1	Hungary 2	Egypt 2
	Colombia 1	Austria 1	Columbia 2
	Poland 1	Columbia 1	Hungary 2
	Slowenia 1	Dominican Republic 1	Austria 1
	Tunesia 1	England 1	Bulgaria 1
		France 1	Dominican Republic 1
		Iran 1	England 1
		Ireland 1	France 1
		Netherlands 1	Iran 1
		Russia 1	Ireland 1
		Serbia 1	Netherlands 1
		Tanzania 1	Poland 1
			Serbia 1
			Slowenia 1
			Syria 1
			Tanzania 1
			Tunesia 1
Other country of birth,mother	Poland 5	Poland 4	Poland 9
	Russia 3	Iran 3	Iran 5
	Türkei 3	Hungary 2	Türkiye 5
	Colombia 2	Kazakhstan 2	Russia 4
	Iran 2	Serbia 2	Austria 3
	Croatia 2	Türkiye 2	Colombia 3
	Austria 1	Algeria 1	Croatia 3
	Belgium 1	Colombia 1	Hungary 3
	Bulgaria 1	Croatia 1	England 2
	China 1	Czech Republic 1	Kazakhstan 2
	Finland 1	Denmark 1	Serbia 2
	Hungary 1	Dominican Republic 1	Spain 2
	Italy 1	Egypt 1	Algeria 1
	Slowenia 1	England 1	Belgium 1
	Spain 1	France 1	Bulgaria 1
	Sweden 1	German Democratic Republic/GDR 1	China 1
	Tunesia 1	Netherlands 1	Czech Republic 1
		Russia 1	Denmark 1
		Slowakia 1	Dominican Republic 1
		South Korea 1	Egypt 1
		Spain 1	Finland 1
		Syria 1	France 1
			German Democratic Republic/GDR 1
			Italy 1
			Netherlands 1
			Slowakia 1
			Slowenia 1
			South Korea 1
			Sweden 1
			Syria 1
			Tunesia 1
Other country of birth,father	Poland 5	Austria 6	Austria 8
	Türkiye 4	Iran 3	Poland 8
	Denmark 3	Poland 3	Türkiye 7
	Russia 3	Türkiye 3	Iran 5
	Austria 2	United States 3	Russia 5
	Croatia 2	Egypt 2	United States 5
	France 2	France 2	France 4
	Iran 2	Italy 2	Denmark 3
	Switzerland 2	Morocco 2	Spain 3
	Slowenia 2	Russia 2	Croatia 2
	United States 2	Serbia 2	Greece 2
	Brazil 1	Spain 2	Italy 2
	Bulgaria 1	Togo 2	Morocco 2
	China 1	Dominican Republic 1	Switzerland 2
	Estonia 1	Greece 1	Serbia 2
	Greece 1	India 1	Slowenia 2
	Moldova 1	Ireland 1	Togo 2
	Netherlands 1	Israel 1	Venezuela 2
	Norwey 1	Latvia 1	Bulgaria 1
	Spain 1	Slowakia 1	Brazil 1
	South Africa 1	South Korea 1	China 1
	Tunesia 1	Syria 1	Czech Republic 1
	Venezuela 1	Czech Republic 1	Dominican Republic 1 Hungary 1
		Hungary 1	India 1
		Venezuela 1	Ireland 1
		Vietnam 1	Israel 1
		Wales 1	Latvia 1
			Moldova 1
			Netherlands 1
			Norwey 1
			Slowakia 1
			South Africa 1
			Syria 1
			Tunesia 1
			Vietnam 1
			Wales 1
Other native language, ^3^	Turkish 3	Arabic 5	Arabic 6
	Russian 3	English 3	Russian 6
	English 2	Russian 3	English 5
	Croatian 2	Spanish 3	Spanish 5
	Polish 2	Farsi 3	Turkish 5
	Spanish 2	Serbian 2	Farsi 4
	Arabic 1	Turkish 2	Polish 3
	Farsi 1	Hungarian 2	Croatian 2
	Mandarin 1	French 1	Serbian 2
		Dutch 1	Hungarian 2
		Polish 1	French 1
		Slovak 1	Mandarin 1
			Dutch 1
			Slovak 1

^1^Listing by frequency, alphabetical if same frequency

^2^Only female and male, there were no patients with diverse sex with migration-related determinants in the evaluation study

^3^Multiple answers were allowed

### Feedback results

Two thirds (66.0%) of the patients found the length of the instrument to be just right regarding the applicability (too long 31.3%; too short 2.6%); 96.3%, respectively found it understandable.

The highest missing rates were seen in the assessment of German language skills in patients with migration-related determinants and in the indication of monthly net equivalent income; for male patients, they were also found in the indication of the highest level of education and current professional activity (see also Tables 2 and 3).

There was a need for clarification and modification of 7 questions on living and income situation, which were commented on at least three times (e.g. “Ultimately share in all of our family everything!” versus distinction according to own income and household desired; “what about child support for children who no longer live in the household?”). The questions on marital status (“I don’t like to be remembered”), children (“living separately”; “sometimes with child”; “living in 2 locations”); household type (“alternative model”); education („A-levels”/ „promotion”) profession (“early retirement”) as well as income (“I don’t see any connection between the other dates and my salary”) were figured out as causing discomfort. Further, the public health institute in Germany (Robert-Koch-Institute, RKI) recommended new minimum indicators for collecting and analyzing migration-related determinants in 2023. The instrument was revised in our interdisciplinary expert panel (Version V1.1).

### Reasons for decline

In the evaluation study, half of the patients, i.e. 49.5% (*n* = 284) declined participation. Seven categories could be formed from the optionally stated reasons for decline (multiple answers were possible; see Additional file 2). The results in Additional file 2 show that the decline rates were similar across recruitment days.

## Discussion

The 6 cognitive interviews led to editorial changes to the instrument. In the evaluation study in 8 departments of a university hospital, the response rate was 50%, with 290 of 574 approached patients participating. Of these patients 96% found the patient instrument understandable. Two thirds considered the length to be appropriate, however, one third found it to be too long. Interestingly, 3% of the patients found the length too short and would have preferred to give more information. Highest missing rates were observed assessing German language skills in patients with migration-related determinants, monthly net equivalent income, and for male patients also regarding the highest level of education and current professional activity. Overall, seven questions on living and income situation (commented at least three times) have shown a need for clarification and modification. The instrument was revised on the patient perspective and patient understanding of the measurements. Non-respondents who optionally provided reasons for their decline in participation in the evaluation study predominantly reported that this was due to insufficient time to answer the instrument in the waiting area setting.

### Identifying barriers for patients in answering sociodemographic questions

It is important to identify, understand and address potential barriers by seeking patient input and views in assessments [31]. As also described by Signorell et al. 2021 in their scoping review, we developed and verified our instrument including expert and patient involvement. We would like to add to the statement by Signorell et al. that “less obvious barriers” may be crucial not only for patient consent to participate in a study in general [31], but also for answering individual study questions, including sociodemographic questions. Our results showed that the question about the patient’s own assessment of their German language skills is difficult to answer, although this question is one of the RKI’s new minimum indicators for collecting migration-related health data. This needs to be clarified and the question might need to be phrased differently. Even then, it should always be remembered that people do not answer because they may feel “monitored” by such questions, because they do not feel secure about their anonymity or because they fear negative consequences due to their possibly “bad” answers. While Signorell et al. 2021 focused on exploring personality traits, we are focusing on taking the patients’ perspective into account by starting with a practical, i.e. sufficiently short, but also sufficiently long and understandable instrument; we hereby incorporated patient feedback to better meet patient needs in long-term practice. In line with Signorell et al. 2021, who pointed out the expert opinion that patients could use their waiting time in the hospital to complete a questionnaire on a voluntary basis during a study visit, we used this approach for the evaluation of our patient instrument.

While the recommendations of the Federal Statistical Office in Germany 2016 optionally differentiated dichotomously between birth in an EU or non-EU country in the assessment, Schenk et al. 2006 provided for an explicit indication of the country of birth of the mother and the father in assessing migration-related determinants in epidemiological research. The country of birth (of the parents) instead of nationality was considered the central indicator for identifying migration-related experience. This was also specified in the data collection of the instrument presented here, according to the national borders applicable at the time of birth. Whereas the new guidelines set out by the German RKI in 2023 recommend the country of birth to be given according to the currently applicable national borders. Recording the specific country of birth of migrants’ parents can provide important contextual information, including cultural background, characteristics of the healthcare system, country-specific mortality and morbidity patterns, and the economic conditions in the country of origin. These factors may continue to shape migrants’ health even after migration [32]. We agree with Schenk et al. 2006 that depending on the research question, a differentiation may be helpful, for example, according to cultural groups, socioeconomic level or medical risk factors. While the question about native language(s) was not answered more often by male than female patients in our study, although this was still the case for less than a quarter of male patients, the question about the German language skills of patients with other native languages was not answered by the majority of patients regardless of whether they were male or female. This could be because it was difficult to choose between good and poor language skills without being able to choose a possible graduation in between. However, none of the participating patients mentioned this as criticism of the instrument in the feedback questions, which leads to the assumption that it could be a problem in general to assess one’s own language skills. The reaction here could also be due to a psychological motive like the answering known as social desirability, only here as an avoidance of answering.

### Patient sociodemographics and standardization in medical reporting

In a 2004 editorial, Lehmann and Miller highlighted that healthcare, unlike other high-risk industries such as aviation, lacks a comparable level of standardization [11]. This deficiency may contribute to variability in care and raise concerns regarding patient safety and quality of care [11]. This also applies to medical research and to the data on which it is based. Despite global efforts of the World Health Organization (WHO) commission on social determinants of health in 2008 [33] the challenge remains, shown by Lynn-Green et al. 2023 [12]. The authors analyzed the reporting of patient sociodemographic data retrospectively in original articles in the NEJM, JAMA, The Lancet, and the AJE from January to December 2020 (*n* = 594) [12]. They described that while sociodemographics in medical reporting in common consist of age along with sex, though the latter being controversial used, other factors like gender identification, race, ethnicity or socioeconomic status (SES) were used inconsistently despite the recent attention [12]. We clearly agree with Lynn-Green et al. 2023 who stated on the variations they found that reports often rely on “binary or limited categories that inadequately capture human diversity” [12].

### The challenge of predictions using sociodemographic data

Bottaro and Faraci 2022 reported that a total of 1046 studies had to be excluded from their systematic review because of the non-use of sociodemographics as predictors [6]. They stated that despite a wide use of sociodemographics in psycho-oncological studies, these variables are often simply used as sample descriptive [6]. We go in line with Lynn-Green et al. 2023 that in medical research the use of sociodemographic data should be more reflected upon [12]. We also agree with Lynn-Green et al. 2023 that pursuing this objective may facilitate the development of more precise research questions aimed at improving the understanding of disease risk and treatment outcomes, ultimately supporting health benefits across diverse populations [12]. Nevertheless, we agree with Lehmann and Miller 2004 that global standardization in health care remains a challenge and has its boundaries underlying the differences in national geography and health policy [9, 11]. And yet, although the standard of care may vary across health care systems due to a variety of factors, researchers should use an optimally standardized approach to collecting patient sociodemographic data to adjust their analyses and calculate predictions.

Regarding SES we agree with Shavers et al. that the choice of the best variable or approach to measure depends in part on its relevance to the population under study as well as the study objective [14]. It is difficult to summarize in short as it consists of a variety of contributing factors. Best practice in reporting SES variables is still missing as Lynn-Green et al. 2023 reported [12]. Despite increasing recognition of the impact of SES parameters in health research, many studies assess socioeconomic status using simplified indicators, often relying on single measures such as categorical variables representing levels of education or income [12]. Regarding this point, we presented and evaluated a detailed yet practical standardization of education as well as income parameters. The recent research of Tetzlaff et al. 2024 revealed a gap in linking German national mortality data with socioeconomic variables [34]. We are in line with the authors who stressed the need to address population inequalities with particular attention to health policy. In our view this also requires emphasizing the importance of medical research in analyzing comparable sociodemographic parameters and identifying risk groups, which includes and goes beyond a comparable determination of socioeconomic disadvantage. Also a systematic analysis of the Global Burden of Disease study 2019 showed the high importance of comparable information on global chronic health burdens for transferring new research findings into clinical applications [35].

### Interpretation and standardization

Bottaro and Faraci 2022 stated that differences in sample size and methods the authors used could have resulted in distortions in the comparison of findings, thus limiting findings. Although they refer to the possibility that the studies’ clinical selection criteria (different tumor sites and disease stages) might be different, they do not mention any potential bias due to possible different underlying variable definitions in compared sociodemographic data. Although Bottaro et al. suggest a more detailed examination of clinical characteristics and at least the inclusion of sociodemographic data, they do not call for standardization of these data. We need clear categories to be able to adequately record patients or test subjects. If these are too narrow, not everyone will be recorded - if they are too broad, the evaluation and generation of results will be difficult. Added to this is the desire for “political correctness” in speech and writing. Just as language is constantly changing, we will also have to adapt the socio-demographic characteristics over time.

### Strengths and limitations

One strength was the plurality of the perspectives of the interdisciplinary expert group. The different professions and experiences in the thematic search for existing patient instruments and their own experiences with the use of instruments in their own projects were reflected in the discourse on the single criteria of the patient instrument on sociodemographics. Another strength was the consensus process modified according to the National Institutes of Health (NIH) type [36]. Briefly, in the first part different members of the interdisciplinary expert panel met in small groups and elaborated common positions of parts of the patient instrument. In the second part the group presents to the plenum the results obtained in the small group discussions. A limitation was that the group discussions in the small groups and in the plenary session were not moderated externally, but care was taken to ensure that all members of the interdisciplinary expert panel agreed to each development step of the instrument. Furthermore, elements of the NIH type were used to reach a consensus [36].

The moderate response rate of 50% could imply a selection bias. For example, there might be a higher proportion of healthier and better educated individuals in our sample than in the general population. Our sample size for the evaluation study was sufficient with 290 cases [25] and we used a two-step design including a small but sufficient number of 6 cognitive interviews covering a heterogenic sample according to age and sex. However, patients were not randomized but consecutively recruited in our evaluation study, and participants with diverse sex turned out to be clearly underrepresented in the sample. Additionally, we would like to point out that some of the patients were unable to fill out the instrument due to no or too little time in the outpatient waiting areas. Also, in general, the results should be replicated in at least one additional sample as recommended by Frost et al. 2007 [25] and confirmatorily validated, which is planned for upcoming revision and future research.

#### Clinical implication and future research

Our research supports Fazal et al. 2023, who concluded that further efforts are required to establish standardized approaches for the collection of sociodemographic data to improve comparability and consistency across studies to advance patient-centered health care [13]. Our goal is therefore not only to address different groups of diseases, but also the different living circumstances of the patients. And we have even started one step earlier, namely with the standardization of the sociodemographic data collected itself. Hereby, the present research focuses on the methodological steps of the development of a patient instrument for sociodemographic data and includes the results of the first two iterative pretests. As a development tool, we have implemented feedback questions to identify specific points for improvement for individual questions. Thus, we go together with Lenzner et al. 2023, who pointed out that cognitive interviews – that is, questionnaire-based semi structured interviews – “cannot be expected to automatically repair questions” in surveys [37]. Like Lenzner et al. 2023, we think that multiple iterative rounds of pretesting are important to ensure higher quality in revised versions than in the first draft [37]. We finally agree with Jones and Hunter that the output developed by a consensus approach “is rarely an end in itself” [23]. Future research will include interval revisions based on identification and discussion of current developments in sociodemographics as well as the use of the instrument in upcoming studies in health services research. Confirmatory factor analyses to validate the German version of the questionnaire, as well as the English-translated version presented in the appendix, constitute topics for future research.

## Conclusion

With our current research, we point out the importance of the comparability of sociodemographic variables in health services research and would like to emphasize the importance of comparable standardization of sociodemographic patient data in studies for stronger support of evidence-based medicine. Our instrument is to be further developed as needed and thus contribute to a well-founded evaluation and analysis of patient data to strengthen evidence-based medicine.

## Supplementary Information

Additional file 1.

Additional file 2.

## Data Availability

Data are not publicly available but can be requested from the corresponding author on reasonable request. Requests for data will be reviewed at the Department of General Practice and Primary Care, University Medical Center Hamburg-Eppendorf. A signed data access agreement with the Department is required before accessing shared data.

## Abbreviations

DESTATIS: Federal Statistical Office of Germany (German Statistisches Bundesamt)
EbM: Evidence-based Medicine
MVZ: Medical Care Center (German Medizinisches Versorgungszentrum)
NIH: National Institutes of Health
OECD: Organisation for Economic Co-operation and Development
RKI: Robert-Koch-Institute
SES: Socioeconomic status
WHO: World Health Organization
