# Supplementary material for: Optimizing patient-reported sociodemographic measures in health care – a national example of the development and evaluation of a practical standardized instrument for collecting patient data in studies
Source: BMC Med Res Methodol. 2026 Aug 20;26:185. doi: 10.1186/s12874-026-02977-z (PMC13491617; doi:10.1186/s12874-026-02977-z)
Supplement: Supplementary file 1 [file 12874_2026_2977_MOESM1_ESM.pdf]

Department of General Practice and Primary Care

Questionnaire for collecting sociodemographic data in studies

Developed by the expert panel on sociodemographic data of the Department of General Practice and Primary Care at the University Medical Center Hamburg-Eppendorf, Germany

Hamburg, 27.11.2023

Version 1.1

1. What is your biological sex?

☐ Female

☐ Male

☐ Diverse

2. In which year were you born?

⌊ ⌋⌊ ⌋⌊ ⌋⌊ ⌋

3. What is your marital status?

☐ Single

☐ Married/registered life partnership

☐ Divorced/life partnership, dissolved

☐ Widowed

4. Do you have biological children or children for whom you share responsibility?

☐ Yes

☐ No

→ 5. If yes, how many?

⌊ ⌋⌊ ⌋ Children

6. Do you live with other people in the same household?
Multiple answers possible.

☐ No, I live alone

☐ Yes, with (spouse) partner

☐ Yes, with child/children

☐ Yes, with own parents or parents of partner

☐ Yes, with other family members

☐ Yes, with other people (e.g. acquaintances, flat-sharing community members, etc.)

7. Which type of household do you live in?

- ☐ Renting in an apartment
- ☐ Renting in a house (single-family house, semi-detached house, terraced house)
- ☐ In a freehold apartment
- ☐ In your own house (single-family house, semi-detached house, terraced house)
- ☐ In assisted living
- ☐ In a retirement or senior citizens' home (largely independent living)
- ☐ In a nursing home (“all-round care”)
- ☐ Other accommodation, namely: ____

8. How many people (including children) live permanently in your household, including yourself?

⎣ ⎦⎣ ⎦ Persons

→ 9. How many of these people are...?

… Under 14 years: ⎣ ⎦⎣ ⎦ Persons

… 14-18 years: ⎣ ⎦⎣ ⎦ Persons

… Over 18 years: ⎣ ⎦⎣ ⎦ Persons

10. What is your highest general school-leaving qualification?

- ☐ No school-leaving qualification
- ☐ Graduation from main school (Hauptschule)/elementary school/8-year elementary school (former GDR before 1965)
- ☐ Secondary school leaving certificate/intermediate school leaving certificate/graduation from secondary school or polytechnic secondary school (POS)
- ☐ Advanced technical college certificate/Fachhochschulreife
- ☐ Abitur/graduation from high school
- ☐ Other school-leaving qualification, namely: ______________________________________

11. What is your highest professional qualification?

- ☐ No professional qualification
- ☐ Vocational-company training (apprenticeship with vocational school)
- ☐ Vocational school education (vocational school, commercial school)
- ☐ specialized school, master school, technical school, vocational academy or technical academy
- ☐ University of Applied Sciences (Bachelor, Master, State Examination, Diploma)
- ☐ University (Bachelor, Master, Magister, State Examination, Diploma)
- ☐ Other professional qualification, namely: ____

12. What is your current professional situation? *Multiple answers possible.*

- ☐ I am a pupil, student, apprentice, or in retraining.
- ☐ I work full-time (minimum of 35 agreed hours per week).
- ☐ I work part-time, on average _____ hours/week.
- ☐ I have a mini-job (income up to a maximum of 520 euros per month).
- ☐ I am self-employed or work as a freelancer.
- ☐ I am a housewife/househusband.
- ☐ I am currently unemployed.
- ☐ I am on permanent sick leave and receive sick pay.
- ☐ I am a retiree or pensioner.
- ☐ Other (e.g. voluntary service, honorary work), please specify:

  ____

13. What is the total monthly net income* available to the people living in your household?

In scientific studies, it is necessary to record economic circumstances, as people with higher and lower incomes can differ significantly in their health. It is therefore important to ask you how much you earn. If you cannot calculate your income exactly, please estimate as best as you can.

- ☐ A Under 700 euros
- ☐ B 700 to under 900 euros
- ☐ C 900 to under 1,100 euros
- ☐ D 1,100 to under 1,300 euros
- ☐ E 1,300 to under 1,500 euros
- ☐ F 1,500 to under 2,000 euros
- ☐ G 2,000 to under 2,500 euros
- ☐ H 2,500 to under 3,000 euros
- ☐ I 3,000 to under 3,500 euros
- ☐ J 3,500 to under 4,000 euros
- ☐ K 4,000 to under 5,000 euros
- ☐ L 5,000 to under 6,000 euros
- ☐ M 6,000 to under 7,500 euros
- ☐ N 7,500 to under 10,000 euros
- ☐ O 10,000 to under 18,000 euros
- ☐ P 18,000 euros and more
- ☐ I cannot answer that

**This refers to the sum of wages, salary, income from self-employment, pension or retirement benefit, each after deduction of taxes and social security contributions. Self-employed persons please state the average net income, minus operating expenses. Please add income from public subsidies, income from renting, leasing, housing benefit, child benefit and other income. Maintenance obligations to others (e.g. ex-partners or children who are not cared for in your own household for at least 50% of the month) must be deducted from this.*

13a. Is there anything we should consider in your answer about income?

- ☐ No
- ☐ Yes, namely: ____

14. How many people in your household live on this income?
Please only include persons who temporarily live in another household (e.g. children with shared parental custody) if they are cared for in your own household for at least 50% of the month.

☐ All persons living in the household

☐ Not all of them, but ⌊ ⌋⌊ ⌋ persons

Of these are ⌊ ⌋⌊ ⌋ persons under 14 years

and ⌊ ⌋⌊ ⌋ persons 14 – 18 years

and ⌊ ⌋⌊ ⌋ persons over 18 years.

☐ I cannot answer that

15. In which country were you born?
Please use the current name of the country, even if the area was previously part of another country. For example: "Czech Republic" instead of "Czechoslovakia".

☐ In Germany

☐ In another country, namely: ____

→ 16. If your country of birth is not Germany: When did you (first) move to Germany?

☐ In the year ⌊ ⌋⌊ ⌋⌊ ⌋⌊ ⌋

17. In which country was your mother born?
Please use the current name of the country, even if the area was previously part of another country. For example: "Czech Republic" instead of "Czechoslovakia".

☐ In Germany

☐ In another country, namely: ____

18. In which country was your father born?
Please use the current name of the country, even if the area was previously part of another country. For example: "Czech Republic" instead of "Czechoslovakia".

☐ In Germany

☐ In another country, namely: ____

19. What nationality(ies) do you have? *Multiple answers possible.*

☐ German citizenship

☐ Other nationality, namely: ____

→ 20. If you do not have German citizenship: What is your current residence status?

☐ I am an EU citizen (EU Freedom of Movement Act)

☐ I have a permanent residence permit

→ → If permanent residence permit: What permanent residence status do you have?

☐ A permanent right of residence in Germany (settlement permit)

☐ A permit for permanent residence in the EU

☐ I have a temporary residence permit

→ → If temporary: What is your temporary residence status?

☐ A temporary residence permit (Aufenthaltserlaubnis)

☐ I am an asylum seeker (Aufenthaltsgestattung)

☐ A toleration (Duldung)

☐ A residence and work permit for highly qualified professionals (EU Blue Card)

☐ Other

21. What language is your native language? *Multiple answers possible.*

☐ German

☐ Other language, namely: ____

→ 22. If your native language is not German: How would you rate your German skills?

☐ Very good

☐ Good

☐ Average

☐ Poor

☐ Very poor

Feedback questions

on the questionnaire for collecting sociodemographic data in clinical studies for patients

1. How do you rate the length of the questionnaire?

☐ Too long

☐ Just right

☐ Too short

2. Are there any questions that you found misleading or incomprehensible?

☐ No

☐ Yes

If yes, which one(s)? (number(s) of question(s)) ________________________________

What exactly was misleading or incomprehensible for you? ____________________

____

3. Are there any questions that were uncomfortable for you to answer?

☐ No

☐ Yes

If yes, which one(s)? (number(s) of question(s)) ________________________________

Would you like to be more specific? ____

____

4. Are there questions for which you were missing additional answer options?

☐ No

☐ Yes

If yes, which one(s)? (number(s) of question(s)) ________________________________

Which answer options were missing for you? __________________________________

____

Do you have additional comments, criticisms or suggestions for improvement?

____

____

Thank you very much for your participation!
