# Supplementary material for: Optimizing patient-reported sociodemographic measures in health care – a national example of the development and evaluation of a practical standardized instrument for collecting patient data in studies
Source: BMC Med Res Methodol. 2026 Aug 20;26:185. doi: 10.1186/s12874-026-02977-z (PMC13491617; doi:10.1186/s12874-026-02977-z)
Supplement: Supplementary file 2 [file 12874_2026_2977_MOESM2_ESM.pdf]

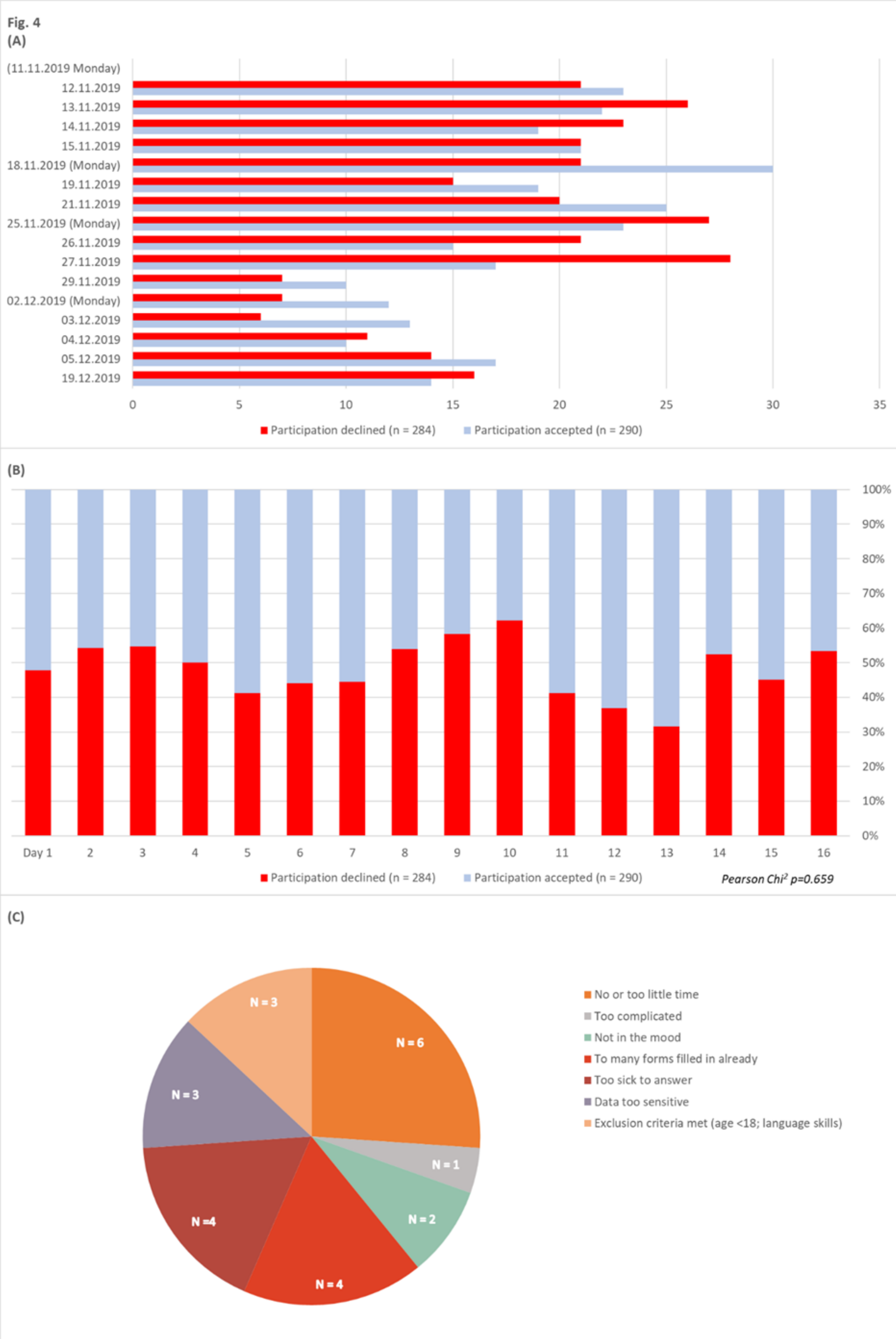

Additional file 2 Data from the recruitment phase regarding participation or decline in the evaluation

study. Absolute (A) and stacked relative (B) frequencies of rejections and acceptances of participation in the evaluation study, which was conducted from November 12th to December 5th, 2019 (recruitment day 1 to 16), as well as the clustered reasons for decline (multiple answers were possible, n = 23 given in total) (C). Two patients gave their written consent but did not return the instrument, thus were counted as non-responders and excluded from further analyses.
